# Rate and risk factors for pediatric cervical spine fusion pseudarthrosis: opportunity for improvement

**DOI:** 10.1007/s43390-023-00641-w

**Published:** 2023-02-06

**Authors:** Edward Compton, Kenneth D. Illingworth, Stephen Stephan, David L. Skaggs, Lindsay M. Andras

**Affiliations:** 1 Children’s Orthopaedic Center, Children’s Hospital Los Angeles, 4650 Sunset Blvd, MS #69, Los Angeles, CA 90027 USA; 2 Cedar-Sinai Medical Center, Los Angeles, CA USA

**Keywords:** Pediatric cervical spine, Nonunion, Occipitocervical fusion, Halo

## Abstract

**Purpose:**

Although the pediatric population typically has a high union rate, the cervical spine has a reputation for frequent pseduarthrosis, as high as 38% in some prior series. Our purpose was to examine the rate and risk factors for pseudarthrosis in pediatric cervical spine fusions.

**Methods:**

Retrospective review of all patients with ≥ 2 years follow-up undergoing cervical spinal fusion between January 2004 and December 2019 at a tertiary pediatric hospital. Pseudarthrosis was defined as an absence of radiographic union as assessed by the attending surgeon for which revision surgery was performed.

**Results:**

64 patients (mean age: 8.4 ± 4.7 years) met inclusion criteria. Mean follow-up was 63.3 ± 41.4 months (range: 24–187 months). 28 fusions (44%) included the occiput. 41 patients (64%) had instrumentation, while 23 patients (36%) had uninstrumented fusions. 48 (75%) patients had a halo for a mean of 97.6 ± 49.5 days. The incidence of pseudarthrosis was as follows: overall = 8/64 (12.5%); posterior fusion = 14.8% (8/54); anterior fusions = 0% (0/4); and anteroposterior fusions = 0% (0/6). The rate of pseudarthrosis was over 8 times higher in fusions involving the occiput (occipitocervical fusion: 25.0%; 7/28 vs. cervical alone: 2.8%; 1/36; *p* = 0.02). Although not statistically significant, the rate of pseudarthrosis was 3 times higher in uninstrumented fusions (21.7%; 5/23) than instrumented fusions (7.3%; 3/41) (*p* = 0.12). In patients with uninstrumented fusion to the occiput, pseudarthrosis rate was 35.7% (5/14), which was higher compared to those who did not (6.0%; 3/50) (*p* = 0.01). Incidence of pseudarthrosis was similar in patients who received autograft (13.0%; 7/54) compared to allograft alone (10.0%; 1/10) (*p* > 0.999).

**Conclusions:**

The pseudarthrosis rate in pediatric cervical spine fusions remained high despite frequent use of halo immobilization and autograft. Patients with uninstrumented occipitocervical fusions are at particularly high risk with more than 1 in 3 developing a pseudarthrosis.

**Study design:**

Retrospective, Comparative.

**Level of Evidence:**

III.

## Introduction

Pediatric cervical spine fusions are indicated for a variety of diagnoses and etiologies [1–4]. Although pediatric spine patients typically have high rates of union, the unique challenges of the pediatric cervical spine make it prone to pseudarthrosis, which has been as high as 38% in prior studies [5]. The unique anatomy and size of the pediatric cervical spine, when compared to adult populations, poses significant challenges in surgical approach and may limit the options for internal fixation [2, 6]. To supplement the often limited fixation options, halo immobilization and autograft may be utilized, but add to the duration of the procedure, as well as the post-operative recovery process. Prior authors have suggested that the use of autograft is imperative to prevent a high pseudarthrosis rate, though this has not been recently examined [7].

Our purpose was to examine the rate and risk factors for pseudarthrosis in the pediatric cervical spine. We hypothesized that uninstrumented fusions and fusions involving the occiput would have a significantly higher rate of pseudarthrosis.

## Materials and methods

Institutional Review Board approval was obtained for this study. Patients < 18 years old with any cervical spine deformity/injury diagnosis and ≥ 2 years of follow-up undergoing a cervical spinal fusion between January 2004 and December 2019 at a tertiary pediatric hospital were retrospectively identified. Electronic medical records, patient imaging, and operative reports were reviewed for patient demographics, cervical spine diagnosis, procedural approach (posterior, anterior, or combined anteroposterior), levels fused/instrumented, type of instrumentation, bone graft type and location of donor site for autografts, use of bone morphogenic protein (BMP), post-operative immobilization method/duration, and complication data. Pseudarthrosis was defined as an absence of radiographic union as assessed by the attending surgeon for which revision surgery was performed. Patients were divided into occipitocervical fusions and fusions not involving the occiput (cervical only fusions).

A Student’s *t* test was used to examine the relationship between means of quantitative outcome variables. A Fisher’s exact test was used to compare categorical variables. Statistical significance was defined as *p* < 0.05. Statistical analysis was performed using STATA/1C 14.0 (Stata Statistical Software:release 14; StataCorp LP, 2015, College Station, TX).

## Results

### Patient characteristics and surgical variables

64 patients met inclusion criteria with a mean age of 8.4 ± 4.7 years (range: 1–17 years). Etiologies included congenital deformities (25), syndromic conditions (14), trauma (13), cervical tumors (7), post-laminectomy kyphosis (4), and neuromuscular scoliosis (1) (Table 1). The mean follow-up duration was 63.3 ± 41.4 months (range: 24–187 months). 67.2% (43/64) patients were male and 32.8% (21/64) of patients were female (Table 1).

**Table 1:** Characteristics, follow-up, and diagnoses of patients undergoing cervical spinal fusion

Characteristic	
Age, y (mean ± s.d.)	8.4 ± 4.7
Sex	
Male	43 (67.2)
Female	21 (32.8)
Height, m (mean ± s.d.)	116.7 ± 29.2
Weight, kg (mean ± s.d.)	32.8 ± 31.4
Follow-up, m (mean ± s.d.)	63.3 ± 41.4
Diagnosis	
Congenital	25 (39.1)
Traumatic	13 (20.3)
Syndromic	14 (21.9)
Spondyloepiphyseal dysplasia	3 (4.7)
Morquio syndrome	2 (3.1)
Down syndrome	3 (4.7)
Larsen syndrome	2 (3.1)
Klippel-Feil syndrome	2 (3.1)
Hurler syndrome	1 (1.6)
Gordon Syndrome	1 (1.6)
Tumor	7 (10.9)
Aneurysmal bone cyst	3 (4.7)
Clival chordoma	3 (4.7)
Osteoblastoma	1 (1.6)
Post-Laminectomy Kyphosis	4 (6.3)
Neuromuscular Scoliosis	1 (1.6)

All values are presented as *n* (%) unless otherwise denoted

s.d. standard deviation

84.4% (54/64) patients had a posterior approach, 9.4% (6/64) had a combined anteroposterior approach, and 6.2% (4/64) had an anterior approach (Table 2). The mean number of levels fused was 3.7 ± 1.6 levels (range: 2–9 levels). 64.1% (41/64) of fusions were instrumented and 35.9% (23/64) were uninstrumented. The occiput was included in the fusion in 43.8% (28/64) of patients. Although not significant, cervical only fusions were instrumented (75.0%; 27/36) more often than fusions that involved the occiput (50.0%; 14/28) (*p* = 0.07). Patients whose fusions involved the occiput (6.5 ± 3.7 years) were significantly younger than patients with cervical fusion alone (9.9 ± 4.9 years) (*p* = 0.004). Of the 28 patients that underwent an occiptocervical fusion, a periosteal turn-down flap was used in 13 (46%) of the patients. No association was found between the use of a periosteal turn-down flap and pseudoarthrosis (*p* = 0.20).

**Table 2:** Surgical variables and immobilization type/duration

Approach, *n* (%)	
Posterior	54 (84.4)
Anterior	4 (6.3)
Anteroposterior	6 (9.4)
Levels fused (mean ± s.d.)	3.7 ± 1.6
Instrumented, *n* (%)	41 (64.1)
Bone graft type, *n* (%)	
Autograft	35 (54.7)
Allograft	10 (15.6)
Autograft/allograft	19 (29.7)
Occipitocervical fusions, *n* (%)	28 (43.8)
Initial immobilization method, *n* (%)	
Halo orthosis	48 (75.0)
Cervical collar	15 (23.4)
Immobilization duration, *d* (mean ± s.d.)	
Halo orthosis	97.6 ± 49.5
Cervical collar	106.3 ± 32.2

Patients with uninstrumented cervical spine fusions (5.5 ± 3.9 years) were significantly younger than patients with instrumented fusions (10.0 ± 4.4 years) (*p* = 0.0001). Patients with uninstrumented occipitocervical fusions (4.7 ± 3.1 years) were significantly younger than all other patients (9.4 ± 4.6 years) (*p* = 0.0007).

### Post-operative immobilization

75.0% (48/64) of patients were initially immobilized with a halo orthosis for a mean of 97.6 ± 49.5 days. 15 of these patients had their halo orthosis removed and were placed into a cervical collar for an additional 65.0 ± 62.0 days. 23.4% (15/64) of patients were initially immobilized with a cervical collar for a mean of 106.3 ± 32.2 days. One patient did not have documentation of the method of post-operative immobilization. There was no significant difference in the total duration of post-operative immobilization between patients initially immobilized with a halo orthosis (120.5 ± 62.4 days) and cervical collar (106.3 ± 32.2 days) (*p* = 0.49).

### Bone grafting

Bone graft was used in all cases. 54.7% (35/64) received autograft alone, 15.6% (10/64) received allograft alone, and 29.7% (19/64) received both allograft and autograft. In total, 54 (84.4%) patients had autograft harvest from another donor site (iliac crest = 46; rib = 8), and 6 of these also had local autograft from the spine. 27/28 (96.4%) patients with occipitocervical fusions received autograft. For patients with cervical fusions alone, 9/36 (25.0%) received allograft alone while 27/36 (75.0%) also had autograft.

Bone morphogenic protein was not used in any of these 64 procedures.

### Pseudarthrosis data

**Fig. 1:**
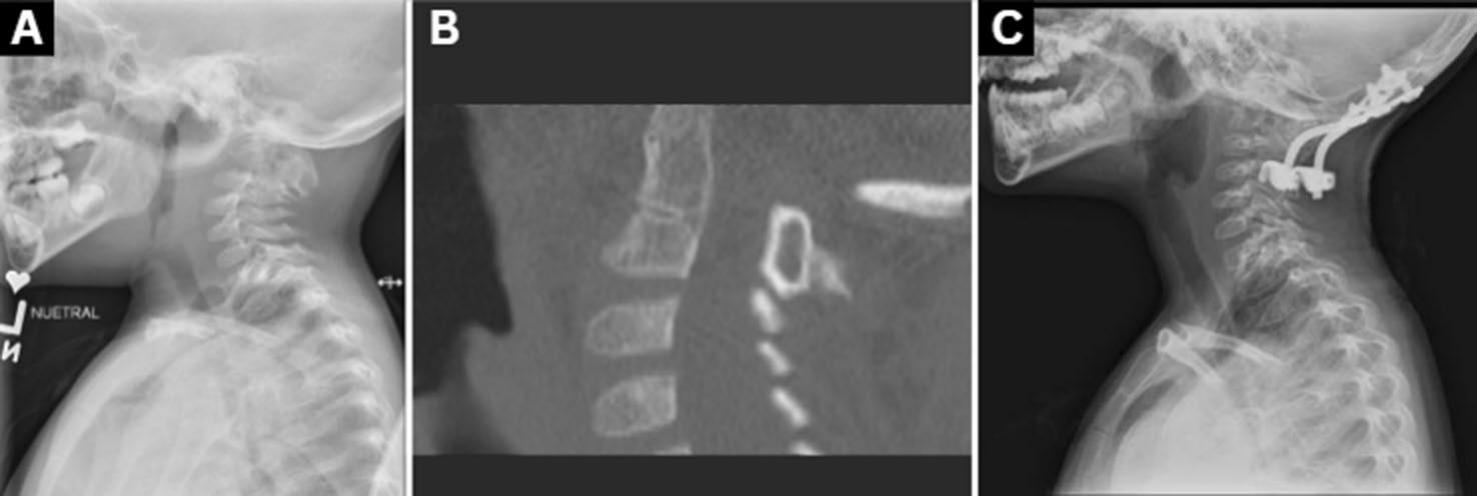
**A** Four year-old male who had 3 previous Chiari decompressions and developed basilar invagination. **B** He initially underwent an attempt at an uninstrumented occipitocervical fusion but developed a nonunion confirmed on CT scan with significant resorption of the graft. **C** This was revised to an instrumented fusion and radiographs with 2 years follow-up confirming an abundant fusion mass

The incidence of pseudarthrosis was as follows: overall = 8/64 (12.5%); posterior fusion = 14.8% (8/54); anterior fusions = 0% (0/4); and anteroposterior fusions = 0% (0/6). Patients with pseudarthrosis are summarized in Table 3 and an example case is demonstrated in Fig. 1. The mean time to pseudarthrosis diagnosis was 188.1 ± 463 days (range: 83–463 days). (Of note, the patient diagnosed with a pseudarthrosis at 83 days had a loss of reduction of their C1-C2 rotatory subluxation that occurred at that time and upon reoperation was noted to have completely resorbed the graft previously placed and did not have any new bone formation.) There was no significant difference in the mean number of levels fused between patients experiencing pseudarthrosis (3.5 ± 0.9 levels) and patients achieving union (3.8 ± 1.6 levels) (*p* = 0.65). The mean age of patients that went on to pseudarthrosis was 6.3 ± 5.0 years compared to 8.7 ± 4.7 years for patients achieving union (*p* = 0.17).

**Table 3:** Nonunion patients

Diagnosis	Time to nonunion (d)	Levels fused	Instrumented (Y/N)	Type of instrumentation
Down Syndrome with atlantoaxial instability	463	OC-C3	Y	Occipital plate, C2 Translaminar Screws, C3 Lateral Mass Screws
C1-C2 Dislocation	140	OC-C2	N	–
Occiput-C2 distraction injury	175	OC-C3	N	–
Basilar Invagination	107	OC-C3	N	–
Atlantoaxial rotatory subluxation	280	OC-C2	N	–
Hemivertebrae with torticollis	127	OC-C4	Y	Occipital plate, C3/C4 lateral mass screws
Traumatic C1-C2 rotatory subluxation	83	C1-C2	Y	C1/C2 Sublaminar Wires
Hemivertebrae with torticollis	130	OC-C2	N	–

Males (8.3 ± 5.0 years) were similar in age to the female patients (8.6 ± 4.1 years) (*p* = 0.76). Male patients (16.3%; 7/43) were not significantly more likely to experience pseudarthrosis than female patients (4.8%; 1/21) (*p* = 0.26). Overall, the rate of pseudarthrosis was 21.7% for uninstrumented fusions (5/23) compared to 7.3% for instrumented fusions (3/41) (*p* = 0.12). The rate of pseudarthrosis was 25.0% for occipitocervical fusions (7/28) vs. 2.8% for cervical only fusions (1/36) (*p* = 0.02). Patients that had an uninstrumented fusion to the occiput (35.7%; 5/14) had a higher rate of pseudarthrosis than those who did not 6.0%; 3/50) (*p* = 0.01) (Table 4).

**Table 4:** Simple logistic regression for risk factors of cervical spinal fusion nonunion

Characteristic	Nonunion, % (n/N)	*p* value
All patients		
Male	16.3 (7/43)	
Female	4.8 (1/21)	0.26
Uninstrumented	21.7 (5/23)	
Instrumented	7.3 (3/41)	0.12
Occipitocervical	25.0 (7/28)	
Cervical only	2.8 (1/36)	0.02*
Uninstrumented occipitocervical	35.7 (5/14)	
All other fusions	6.0 (3/50)	0.01*
Autograft	13.0 (7/54)	
Allograft alone	10.0 (1/10)	> 0.99
Halo	14.6 (7/48)	
Cervical collar	6.7 (1/15)	0.67
Cervical only		
Autograft	0 (0/27)	
Allograft alone	11.1 (1/9)	0.25

*indicates significance at the *p* < 0.05 level

Overall, there was a similar incidence of pseudarthrosis in the patients who received autograft (13.0%; 7/54) to those who received allograft alone (10.0%; 1/10) (*p* > 0.999). As all but one of the occipitocervical fusions received autograft, no subanalysis could be performed for those patients related to bone graft. For the patients with cervical fusions alone, 11.1% (1/9) of patients who received allograft alone and 0% (0/29) of patients who had autograft placed developed a pseudarthrosis (*p* = 0.25). There was also a similar incidence of pseudarthrosis in patients initially immobilized with a halo (14.5%; 7/48) versus cervical collar (6.7%; 1/15) (*p* = 0.67).

For the patients that experienced a pseudarthrosis, 5 returned to the OR for revision of instrumentation, 2 for bone grafting alone, and 1 for revision of the halo. No patient had evidence of infection upon return to the OR for management of the pseudarthrosis. Seven of the eight patients went on to heal, however 1 patient was lost to follow-up.

In addition to the return to operating room for management of a pseudarthrosis, 8 patients also underwent additional surgery for the following complications: implant failure (3), progressive deformity above/below the level of the previous fusion (2), residual cord compression (1), dural tear repair (1), and implant prominence (1). The three cases of implant failure consisted of 1 rod disengagement, and 2 incidences of anchor pullout.

## Discussion

Pediatric cervical spine fusions may be indicated for a variety of traumatic, tumor, congenital or syndromic causes [1–4]. The small and  often aberrant anatomy poses significant challenges in surgical management of these patients. [2, 6]. Although pediatric patients typically have high rates of union, the unique challenges of the pediatric cervical spine make it prone to pseudarthrosis, which has been reportedly as high as 38% in prior studies [5]. The present study of 64 patients was used to evaluate the rate and risk factors for pediatric cervical spinal fusion pseudarthrosis.

The overall rate of pseudarthrosis was 12.5%, with all pseudarthrosis occurring in isolated posterior spinal fusions. On further analysis, we found trends toward a higher rate of pseudarthrosis in uninstrumented fusions vs. instrumented fusions (21.7% vs. 7.3%) and in occipitocervical fusions vs. cervical only fusions (25.0% vs. 2.8%), though in isolation only occipitocervical vs cervical only fusions achieved significance (*p* = 0.12 and 0.02, respectively). The lack of significance in uninstrumented vs instrumented fusions as a single variable may be attributable to the study being underpowered, though further study is needed to confirm this. However, in combination, patients that had an uninstrumented fusion to the occiput (35.7%; 5/14) had a higher rate of pseudarthrosis than those who did not (6.0%; 3/50) (*p* = 0.01). The fact that more than a third of the patients with uninstrumented occipitocervical fusions developed pseudarthrosis certainly gives one pause. Of note, these pseudarthrosis occurred despite frequent use of halo immobilization and autologous bone graft harvest as precautionary measures.

In a prior study of occipitocervical fusions, He et al*.* examined 54 uninstrumented fusions and 262 instrumented fusions in patients aged 14–71 years old [8]. In the uninstrumented fusion group, the authors found 7 patients who did not achieve successful union. In the instrumented fusion group, there was only 1 case of delayed bone-graft fusion. The authors concluded that in occipitocervical fusions, there is a high rate of complications, especially if no instrumentation is used [6]. Although the age group differed significantly in our series, our findings corroborate the findings by He et al*.* In the pediatric population, a prior study of 25 patients undergoing upper cervical or occipitocervical fusions with a wiring technique by Lowry et al*.* reported 4 pseudarthrosis (16%), with 3 more patients achieving union after a revision procedure. The authors attributed the lack of fusion to inadequate post-operative immobilization, which may have resulted in intersegmental micromotion. They recommended immobilization until a solid fusion mass is visualized.

Other studies have attributed the high pseudarthrosis rate to the type of instrumentation. In a study of 20 pediatric patients undergoing cervical spinal fusion, Mitchell et al*.* reported on 15 patients in which both of the patients who developed a pseudarthrosis had wiring alone as fixation. The authors concluded that cervical spine wiring is associated with a high risk for pseudarthrosis leading to revision procedures and that rigid segmental instrumentation with screw-based constructs may be superior for avoiding pseudarthrosis. A review by Hwang et al*.* examined 285 occipitocervical fusions and 181 fusions below the occipitocervical junction in pediatric patients less than 18 years old [2]. In occipitocervical fusions, they found a 99% fusion rate in screw-based constructs versus only 95% in wire-based constructs, although this difference was not significant. Likewise, in fusions below the occipitocervical junction, they found screw-based constructs had a 99% fusion rate versus only 83% with wiring (*p* < 0.05). They concluded that screw-based constructs, even with lower rate of halo immobilization, may lead to high fusion rates. Of note, many studies in their series used adjuvant materials such as bone morphogenic protein and demineralized bone matrix, but their use was inconsistently reported, so further analysis could not be done on adjuvant materials. Of the 8 pseudarthrosis in our series, only 3 occurred following instrumented fusions. Of these, 1 was in a case below the occipitocervical junction in which a wiring technique was used. Although this study is underpowered to determine a difference between types of instrumentation, our findings are in line with the observations of Hwang’s series that wire-based constructs below the occipitocervical junction may lead to increased rates of pseudarthrosis.

We observed a high rate of pseudarthrosis in patients with occipitocervical fusions despite harvesting autograft. Dormans et al*.* recommended use of autograft in cervical fusions in pediatric patients, and as this series demonstrates our surgeons have generally followed that recommendation with 84% receiving autograft [7]. Although only 12 patients (16%) had allograft alone, 92% of these went on to union. This may represent some selection bias in the surgeon’s decision not to harvest autograft. However, given the high rate of union in the allograft only cases, specifically in cervical only fusions, this appears to be a reasonable choice. Given the high rate of pseudarthrosis in occipitocervical fusions despite use of autograft in all but one case, it remains to be determined whether the additional graft harvest was beneficial to these patients or not. Further investigation on this topic is undoubtedly warranted and is being discussed as a multicenter study, but is a question that will likely take many years to answer.

This study is limited by its retrospective nature. Additionally, defining pseudarthrosis may be challenging in this area as a clear universal radiographic definition is lacking. Attempts at relying on radiology reports were unsuccessful as many omitted any commentary on the fusion mass (or lack thereof). Recently, we have transitioned to getting more limited CT scans to evaluate this, but even with that some are difficult to interpret due to metal artifact. Consequently, we used revision surgery as a more clinically relevant parameter. It is certainly possible that this underestimated the true incidence of pseudarthrosis, and the issue is even larger than it appears here, though given the mean duration of follow-up was over 5 years, it seems likely that if this was underappreciated the majority would have developed additional issues that presented themselves within that time frame. Furthermore, if the pseudarthrosis rate was in fact even higher than reported here that in some ways only further emphasizes the conclusion that particular caution needs to be used in these cases.

Although this is a large series of pediatric cervical spinal fusions compared to many of the prior publications on this topic, the sample size still has limitations, particularly for sub analysis. A large prospective database of multiple centers is in the formative stages to try to address some of the questions that this study was underpowered to answer but will be many years in the making. Nevertheless, this study provides ample evidence that the pseudarthrosis rate in cervical spine fusions, 12.5% overall in this series, is high compared to most pediatric procedures. Additionally, all but one of these occurred in the occipitocervical fusion group (7/28; 25%) vs the cervical fusion only group (1/36; 2.8%). This is of significance both statistically (*p* = 0.02) and clinically, as this appears to be predominantly an issue with occipitocervical fusions. Furthermore, the use of autograft, instrumentation and halo immobilization in these cases undoubtedly reflects some selection bias. However, this series highlights the challenges of achieving stable fusion in this setting.

In conclusion, the pseudarthrosis rate in pediatric cervical spine fusions remained high despite frequent use of halo immobilization and autograft. Patients with uninstrumented occipitocervical fusions are at particularly high risk with more than 1 in 3 developing a pseudarthrosis.

## Key points

- The overall rate of pseudarthrosis in pediatric cervical spinal fusions was 12.5%.
- The pseudarthrosis rate in pediatric cervical spinal fusions remained high despite frequent use of halo immobilization and autograft.
- Patients with uninstrumented occipitocervical fusions are at particularly high risk of pseudarthrosis, with more than 1 in 3 developing a pseudarthrosis.
- In cervical only fusions, allograft may be adequate for grafting as there was a similar incidence of pseudarthrosis in this subset of patients both with and without autograft although the numbers were small.

## Data Availability

Datasheet can be provided by contacting the corresponding the author.

## Declarations

### Human and animal resources

Additional declarations for articles in life science journals that report the results of studies involving humans and/or animals: Not applicable.
